# Intrinsic functional connectivity correlates of cognitive deficits involving sustained attention and executive function in bipolar disorder

**DOI:** 10.1186/s12888-023-05083-2

**Published:** 2023-08-11

**Authors:** Yan-Kun Wu, Yun-Ai Su, Lin-Lin Zhu, Ji-Tao Li, Qian Li, You-Ran Dai, Jing-Yu Lin, Ke Li, Tian-Mei Si

**Affiliations:** 1 Peking University Sixth Hospital, Peking University Institute of Mental Health, NHC Key Laboratory of Mental Health (Peking University), National Clinical Research Center for Mental Disorders (Peking University Sixth Hospital), Beijing, 100191 China; 2 PLA Strategic Support Force Characteristic Medical Center, Beijing, 100101 China

**Keywords:** Magnetic resonance imaging, Bipolar disorder, Default mode network, Executive function, Attention

## Abstract

**Background:**

The neural correlate of cognitive deficits in bipolar disorder (BD) is an issue that warrants further investigation. However, relatively few studies have examined the intrinsic functional connectivity (FC) underlying cognitive deficits involving sustained attention and executive function at both the region and network levels, as well as the different relationships between connectivity patterns and cognitive performance, in BD patients and healthy controls (HCs).

**Methods:**

Patients with BD (*n* = 59) and HCs (*n* = 52) underwent structural and resting-state functional magnetic resonance imaging and completed the Wisconsin Card Sorting Test (WCST), the continuous performance test and a clinical assessment. A seed-based approach was used to evaluate the intrinsic FC alterations in three core neurocognitive networks (the default mode network [DMN], the central executive network [CEN] and the salience network [SN]). Finally, we examined the relationship between FC and cognitive performance by using linear regression analyses.

**Results:**

Decreased FC was observed within the DMN, in the DMN-SN and DMN-CEN and increased FC was observed in the SN-CEN in BD. The alteration direction of regional FC was consistent with that of FC at the brain network level. Decreased FC between the left posterior cingulate cortex and right anterior cingulate cortex was associated with longer WCST completion time in BD patients (but not in HCs).

**Conclusions:**

These findings emphasize the dominant role of the DMN in the psychopathology of BD and provide evidence that cognitive deficits in BD may be associated with aberrant FC between the anterior and posterior DMN.

## Background

Although mood instability is the core characteristic of bipolar disorder (BD), deficits in cognitive performance are also common but underestimated. Cognitive deficits exist persistently across acute episodes and euthymic states and may track illness progression independently of illness severity [1]. However, reliable treatments targeted at cognition are lacking [2], which is partially attributed to limited insight into the neurobiological underpinnings of cognitive deficits. The neural correlate of cognitive deficits is an issue of profound clinical and research interest that warrants further investigation.

The default mode network (DMN), central executive network (CEN) and salience network (SN) are three core neurocognitive networks that have been identified in the human brain in terms of their important roles in higher cognition [3]. The CEN is crucial for supporting executive functions, such as working memory and problem solving, whereas the DMN is responsible for episodic memory retrieval and social cognitive processes [3]. The SN serves as a bridge between the external-oriented CEN and internal-oriented DMN by detecting and mapping external stimuli and internal mental events [4]. Neuroimaging studies have demonstrated that functional connectivity alterations in these three brain networks are implicated in mood dysregulation in BD [5–8], but there are relatively few studies that have investigated the cognitive implications of these brain networks.

Commonly observed cognitive deficits in BD are related to the domains of executive function and sustained attention [9]. Several task-related functional imaging studies have supported the idea that aberrant activations in the DMN, CEN and SN [10, 11] play a pivotal role in cognitive deficits in BD. For example, hypoactivation in the dorsolateral prefrontal cortex (dlPFC) and the parietal cortex in the CEN may elicit working memory dysfunction [12–14]. Aberrant activation in the ventrolateral prefrontal cortex (vlPFC) [15, 16] and insula [11, 17] in the SN is associated with response inhibition and sustained attention dysfunction, respectively. In addition, resting-state functional connectivity (FC) offers an opportunity to examine the intrinsic activity of neural circuitry associated with cognitive deficits. A negative association was observed between inferior temporal gyrus-dorsal caudal putamen FC and the ability to discriminate a signal (target) from background noise (the nontarget) [9], whereas a positive association was found between perigenual anterior cingulate cortex (ACC)-related FC and sustained attention function [18]. However, another study did not find any significant correlation between inferior frontal gyrus (IFG) seed-based FC and visual sustained attention performance [19]. The contradictory findings related to the relationship between resting-state FC and cognitive measure likely due to differences in neuropsychological tests used, the cognitive domains explored, technical issues related to measuring FC, etc. [20].

Despite this progress in neural correlates underlying cognitive deficits, there are several limitations to the literature. For example, there are relatively few studies examining how brain regions interplay in a temporal manner to inform executive function performance in the absence of task demands, with a particular lack of data that simultaneously assess executive function and sustained attention [9]. Furthermore, although the aberrant activations in the brain network suggest that the intrinsic network may exhibit altered FC with brain regions, relatively few studies have investigated the altered functional connectivity at both the region and network levels. Finally, many studies have employed simple correlation analyses to explore the relationship between FC and cognitive performance solely in BD patients. An alternative strategy is to identify different relationships between connectivity patterns and cognitive performance between BD patients and healthy controls (HCs) using linear regression analyses by examining the interaction effect of diagnosis group and FC on cognitive performance [21]. This strategy may help to identify the unique FC correlates of cognitive deficits involving sustained attention and executive function in BD, which are not observed in HCs [22].

In this study, we first examined cognitive deficits related to sustained attention and executive function in BD. Subsequently, we explored the resting-state functional connectivity of the DMN, CEN and SN at both the region and network levels in BD patients compared with HCs. Finally, we examined the different connectivity patterns associated with cognitive deficits in executive function and sustained attention in BD patients compared with HCs. We hypothesized that BD patients would have altered FC at both the region and brain network levels and may be related to worse cognitive performance in BD patients.

## Methods

### Participants

Seventy-nine bipolar disorder participants were recruited from the Outpatient Department of Peking University Sixth Hospital. Diagnoses were confirmed according to the DSM-IV-TR criteria for BD by two qualified psychiatrists using the Mini-International Neuropsychiatric Interview (M.I.N.I.) [23]. All of the BD patients met the DSM-IV-TR criteria for bipolar disorder. BD patients with comorbid DSM-IV-TR Axis I disorder (except for anxiety disorders), patients with an Axis II personality disorder or intellectual disability and patients with a diagnosis of the rapid cycling disorder subtype or current mixed episode were excluded from the study. Fifty-six HCs (matched by sex and age) were recruited from the local community via offline and online advertisements and word-of-mouth. HCs with a personal (screened by two qualified psychiatrists using the M.I.N.I.) or family history (documented by self-reported measure) of any psychiatric disorder or with a history of psychotropic drug use were excluded from the study. All of the participants were between the ages of 18 and 55 years and were right-handed. Exclusion criteria for all of the participants included serious physical illness, current pregnancy or breastfeeding, alcohol/substance misuse in the last 12 months, electroconvulsive therapy in the last six months, acute suicidal ideations or behaviours and any contraindications to magnetic resonance imaging (MRI) scanning. The study was approved by the independent Ethics Committee of Peking University Sixth Hospital, and written informed consent was obtained from all of the participants before data collection. All procedures contributing to this work comply with the ethical standards of the relevant national and institutional committees on human experimentation and with the Helsinki Declaration of 1975, as revised in 2008.

### Clinical and neuropsychological assessment

Symptom severity was assessed among patients by using the 17-item Hamilton Rating Scale for Depression (HRSD-17) [24] and Young Mania Rating Scale (YMRS) [25]. A self-report scale known as the Positive And Negative Affect Schedule (PANAS) [26], which contains 10 positive affect (PA) and 10 negative affect (NA) items, was administered to assess the opposing valences of affect in the current mood state.

Cognitive function in attention/vigilance and executive function domains was assessed among all of the participants by using computerized neuropsychological tests. The continuous performance test (CPT) [27] and the Wisconsin Card Sorting Test (WCST) [28] were administered to all of the participants. Additionally, the neuropsychological assessment was administered by using standard instructions in a quiet testing room on the day before scanning.

### MRI data acquisition and processing

Participants were scanned on a 3-Tesla Siemens Trio MR scanner in the 306th Hospital. The functional images lasted seven minutes by using the T2*-weighted gradient-echo echo-planar imaging sequence with the following parameters: repetition time = 2000 ms; echo time = 30 ms; in-plane resolution of 3.3 × 3.3 mm^2^; matrix of 64 × 64; field of view of 210 × 210 mm^2^; flip angle of 90^°^; 30 slices; 210 volumes; and a thickness/gap of 4.0 mm/0.8 mm. The structural images were obtained by using a T1-weighted, magnetization-prepared, rapidly acquired gradient-echo (MPRAGE) sequence with the following parameters: repetition time = 2300 ms; echo time = 3.01 ms; in-plane resolution of 1.0 × 1.0 mm^2^; matrix of 256 × 256; field of view of 240 × 256 mm^2^; flip angle of 9^°^; 176 slices; and a thickness of 1.0 mm. During the resting-state scanning procedure, participants were instructed to relax with their eyes closed, to avoid head motion and to not fall asleep. A simple questionnaire was performed to confirm that the participants had followed the instructions. Only eligible participants were included in the study.

The MRI data were preprocessed via the DPABISurf toolbox (DPABISurf_V1.2_190919, http://rfmri.org/DPABISurf). After removing the first 10 volumes, the remaining 200 volumes were corrected for different slice acquisition times and realigned to correct for inter-TR head motion. Nuisance covariates were regressed out from the time series, including the Friston-24 parameters of head motion, timeseries extracted from cerebrospinal fluid regressors and white matter regions and linear trends. Moreover, the structural images were segmented by using fast (FSL 5.0.9) [29] and normalized to the Montreal Neurological Institute (MNI) space by using antsRegistration (ANTs 2.2.0). The derived functional images were coregistered to the corresponding structural images, normalized to the MNI space with the warping parameters and resampled to 3 mm cubic voxels. Subsequently, the images were bandpass filtered (0.01–0.1 Hz) and spatially smoothed by using a 6 mm full width at half maximum. To minimize head motion effects on the data, a rigorous threshold for mean framewise displacement (FD) (no larger than 0.2 mm) was applied. Twenty BD patients and four HCs were excluded from the analyses for excessive head motion during scanning. Therefore, the analysis was performed with the remaining dataset comprising 59 BD patients and 52 HCs.

### Seed-based functional connectivity analysis

We conducted a seed-to-whole-brain functional connectivity analysis by using key hubs of the three brain networks as seeds. Key hubs included bilateral posterior parietal cortex and bilateral dlPFC for the CEN, bilateral posterior cingulate cortex (PCC) and bilateral ventromedial prefrontal cortex (vmPFC) for the DMN and bilateral vlPFC and bilateral dorsal ACC (dACC) for the SN [30]. Each seed comprised a 5-mm-radius spherical ROI. The MNI coordinates (x, y, z) of the centre of the spheres were listed as follows: i) right dlPFC (46, 46, 14) , left dlPFC (-34, 46, 10), right lateral parietal cortex (38, − 56, 44) and left inferior parietal lobule (IPL) (− 48, − 48, 48) in the CEN, ii) bilateral vmPFC (± 2, 36, 10) and bilateral PCC (± 7, 36, 10) in the DMN and iii) right dACC (6, 22, 30), left dACC (− 6, 18, 30), left vlPFC (− 38, 52, 10) and right vlPFC (42, 46, 0)  in the SN [30]. The mean time series of blood oxygenation level-dependent (BOLD) signals were extracted from each seed. Functional connectivity values were defined as the Pearson’s correlation coefficients between the mean time series of each seed and that of each voxel in the whole brain. Afterwards, the FC values were transformed to z scores by using the Fisher r-to-z formula.

We performed a two-sample *t* test with age, sex, years of education and mean FD values as covariates in the FC maps between BD patients and HCs to evaluate the group differences. Gaussian random field (GRF) theory was employed to correct for multiple comparisons with a height threshold of Z > 3.1 and a cluster probability of *p* < 0.05. The family wise error rate could be controlled under 5% [31] with this method.

### Relationship between functional connectivity and neuropsychological assessments

To examine the different relationships between functional connectivity and cognitive performance between BD patients and HCs, we included functional connectivity related to the three core neurocognitive networks that showed significant group differences (totally 30 pairs of functional connectivity) in the separate multiple regression analyses as the independent variable, with each of the neuropsychological assessments showing significant group differences (totally 4 measures) as the dependent variable. We entered FC value, diagnostic group, FC-by-diagnostic-group interaction term, age, sex, education years and head motion as predictors. Moreover, we entered one pair of functional connectivities that showed significant group comparison results at a time. We focused on the neuropsychological measure that showed a significant group-by-FC interaction effect and performed post hoc analysis in BD patients and HCs, respectively.

### Sensitivity analysis

To determine whether the changes in functional connectivity present in euthymic state, we extracted the FC values in significant clusters related to the three core neurocognitive networks and performed a two-sample *t* test with age, sex, years of education and mean FD values as covariates between euthymic patients with BD and HCs for each significant cluster. False discovery rate (FDR) correction was applied to control for multiple comparisons.

To examine the impact of current mood state on relationship between FC and neuropsychological assessments in BD patients, two exploratory analyses were conducted in the neuropsychological measure that showed a significant interaction effect of diagnostic group and FC. One post hoc analysis employing multiple regression model was conducted in euthymic patients with BD and acute patients with BD, respectively. Another post hoc analysis employing multiple regression model was conducted in all BD patients with current mood state (i.e., depression, (hypo)mania and euthymia) as one of predictors.

To control the potential medication effect in BD patients, a subsidiary analysis was conducted by entering a binary variable indicating whether on treatment or not as one of predictors.

## Results

### Demographic, clinical and cognitive information

Complete participant demographic and clinical characteristics are presented in Table 1. No significant differences were found between the BD patients and HCs in age or sex. HCs had more years of education than BD patients. Regarding the WCST, although BD patients had comparable performance compared with HCs, BD patients spent a longer time finishing the test after controlling for years of education. In the attention/vigilance domain, BD patients displayed significantly poorer performance than HCs after controlling for years of education. Specifically, BD patients had fewer total correct responses and more omissive responses, and they also spent a longer time on the CPT.

**Table 1:** Demographics and clinical characteristics of the subjects

		**BD** **(*n* = 59)**	**HCs** **(*n* = 52)**	***p***	**Effect size**
Sex, female (n [%])		37 (62.7)	39 (75.0)	0.16^a^	N/A
Age (years, mean [SD])		27.64 (8.46)	27.69 (9.30)	0.98^b^	N/A
Education (years, mean [SD])		14.73 (3.43)	17.00 (1.56)	< 0.001^b^	N/A
YMRS score (mean [SD])		3.62(7.46)	N/A	N/A	N/A
HRSD score (mean [SD])		9.71(9.60)	N/A	N/A	N/A
PANAS score (mean [SD])		50.88(11.54)	N/A	N/A	N/A
PANAS-PA score (mean [SD])		24.81(8.11)	N/A	N/A	N/A
PANAS-NA score (mean [SD])		26.07(9.31)	N/A	N/A	N/A
Mood Stabilizers (n [%])		19 (32.2)^d^	N/A	N/A	N/A
Antidepressants (n [%])		16 (27.1)^d^	N/A	N/A	N/A
Antipsychotics (n [%])		23 (39.0)^d^	N/A	N/A	N/A
Benzodiazepines (n [%])		0 (0)^d^	N/A	N/A	N/A
Unmedicated (n [%])		13 (22.0)	N/A	N/A	N/A
Type I BD (n [%])		38 (64.4)	N/A	N/A	N/A
Current mood state (n (%))	Depression	25 (42.4)	N/A	N/A	N/A
	(Hypo) mania	9 (15.2)	N/A	N/A	N/A
	Euthymia	25(42.4)	N/A	N/A	N/A
WCST	Percentage of Error Responses (mean [SD])	0.313 (0.14)	0.272 (0.15)	0.57^c^	< 0.01^e^
	Percentage of Perseverative Responses (mean [SD])	4.58 (3.68)	6.81 (10.89)	0.87^c^	< 0.01^e^
	Percentage of Nonperseverative Error Responses (mean [SD])	0.268 (0.15)	0.207 (0.14)	0.25^c^	0.01^e^
	Percentage of Conceptual Level Responses (mean [SD])	0.650 (0.18)	0.707 (0.17)	0.40^c^	< 0.01^e^
	Completion time, (s, mean [SD])	401.29 (236)	263.62 (104)	0.002^c^	0.09^e^
CPT	Total correct responses (mean [SD])	98.76 (16.27)	110.15 (6.51)	0.001^c^	0.10^e^
	Total error response (mean [SD])	4.87 (8.89)	1.81 (1.35)	0.17^c^	0.02^e^
	Total omissive responses (mean [SD])	19.86 (13.18)	9.83 (6.43)	< 0.001^c^	0.11^e^
	Completion time (s, (mean [SD])	7372 (923)	6653 (813)	0.001^c^	0.09^e^

*HRSD* Hamilton Rating Scale for Depression, *YMRS* Young Mania Rating Scale, *PANAS* Positive And Negative Affect Schedule, *WCST* Wisconsin Card Sorting Test, *CPT* Continuous Performance Test

^a^Chi-square test

^b^two-sample *t* test

^c^two-sample *t* test controlling for education years

^d^eleven patients’ medication data were missing

^e^effect size was calculated via *η*^*2*^

### Functional connectivity analyses

**Fig. 1:**
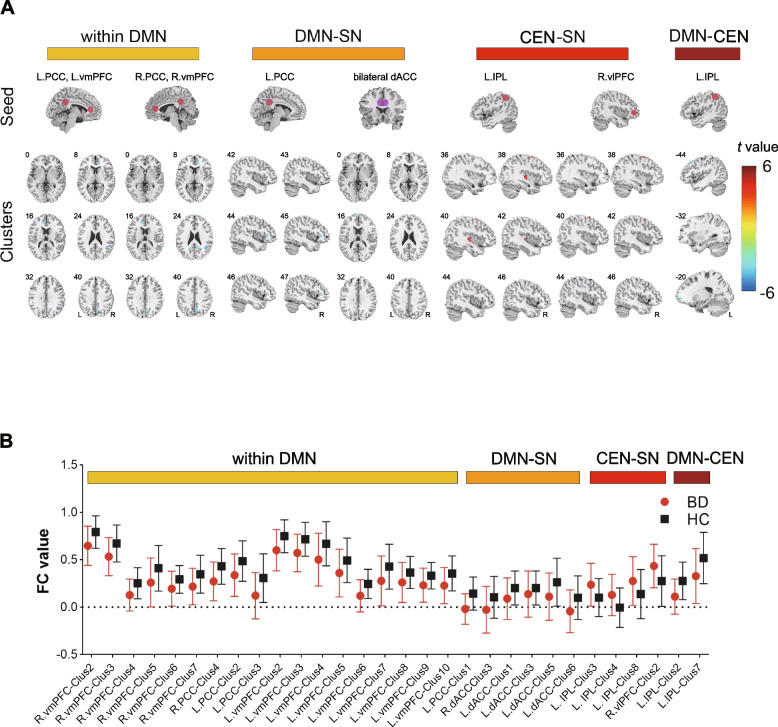
Group effect on the FC profiles related to the three core neurocognitive networks in BD patients and HCs. **A** The brain maps show the clusters with significant between-group differences in FC profiles. The panel labelled ‘within DMN’ shows the 18 clusters exhibiting significant FC with seeds in the DMN. The panel labelled ‘DMN-SN’ shows one cluster in SN exhibiting significant FC with the left PCC and the five clusters in the DMN showing significant FC with the bilateral ACC. The panel labelled ‘CEN-SN’ shows the three clusters in SN exhibiting significant FC with the left IPL and one cluster in CEN showing significant FC with the right vlPFC. The panel labelled ‘DMN-CEN’ shows the two clusters in the DMN exhibiting significant FC with the left IPL. **B** The symbol plot shows the mean FC value (symbols) and standard deviation (upper and lower lines). X axis labelling refers to corresponding functional connectivity in Table 2

The analyses revealed 45 clusters with significant group differences in FC with the seeds. Thirty of these regions were located in the CEN, DMN and SN (Fig. 1A).

### ROI-wise connections

For seeds in the CEN, only the left IPL showed significant FC differences in FC in BD patients. BD patients exhibited increased FC between the left IPL and right insular/left central opercular cortex/right paracentral lobule in the SN but decreased FC between the left IPL and left orbital part of the superior frontal gyrus (SFG)/left middle frontal gyrus (MFG) in the DMN (Table 2).

**Table 2:** Functional connectivity differences between BD patients and HCs

Seed	Target network	Cluster	Clusters which showed significant FC with seed	Cluster size (voxels)	MNI coordinates (x, y, z)	Peak *t* value	Network
**L. IPL**	CEN	Clus 1	R. Fusiform	38	42.5, -16.5, -26.5	4.01	SCN
		Clus 2	L. Frontal_Sup_Orb	21	-17.5, 53.5, -4.5	-4.35	DMN
		Clus 3	R. Insula	43	40.5, -18.5, 3.5	4.08	SN
		Clus 4	L. Rolandic_Oper	20	-49.5, 1.5, 9.5	3.90	SN
		Clus 5	L. Occipital_Mid	81	-33.5, -70.5, 3.5	3.97	VN
		Clus 6	L. SupraMarginal	52	-53.5, -22.5, 19.5	3.92	SMN
		Clus 7	L. Frontal_Mid	38	-45.5, 21.5, 45.5	-3.96	DMN
		Clus 8	R. Paracentralobule	97	16.5, -44.5, 49.5	4.04	SN
		Clus 9	L. Postcentral	22	-39.5, -42.5, 65.5	3.63	SMN
**R. vmPFC**	DMN	Clus 1	R. Fusiform	37	42.5, -16.5, -24.5	-4.14	SCN
		Clus 2	R. Cingulum_Ant	58	10.5, 35.5, 7.5	-4.28	DMN
		Clus 3	L. Cingulum_Ant	82	-5.5, 37.5, 15.5	-4.15	DMN
		Clus 4	R. Angular	83	46.5, -50.5, 21.5	-4.79	DMN
		Clus 5	R. Frontal_Sup_Medial	45	4.5, 65.5, 23.5	-4.39	DMN
		Clus 6	L. Precuneus	20	-15.5, -54.5, 25.5	-4.02	DMN
		Clus 7	R. Precuneus	93	6.5, -62.5, 39.5	-3.98	DMN
**R. PCC**	DMN	Clus 1	Vermis_10	28	2.5, -46.5, -26.5	-4.04	
		Clus 2	L. Cerebelum_Crus2	68	-5.5, -82.5, -20.5	-4.80	
		Clus 3	R. Pallidum	47	14.5, -2.5, -2.5	-4.36	
		Clus 4	L. Cingulum_Ant	21	-11.5, 39.5, -2.5	-4.41	DMN
**L. PCC**	DMN	Clus 1	R. Frontal_Inf_Tri	23	44.5, 29.5, -0.5	-3.92	SN
		Clus 2	R. Cingulum_Ant	47	10.5, 47.5, 9.5	-3.96	DMN
		Clus 3	R. Temporal_Sup	68	64.5, -46.5, 19.5	-4.19	DMN
**L. vmPFC**	DMN	Clus 1	R. Fusiform	38	42.5, -18.5, -22.5	-4.16	SCN
		Clus 2	R. Cingulum_Ant	61	10.5, 35.5, 7.5	-4.36	DMN
		Clus 3	L. Cingulum_Ant	117	-13.5, 39.5, 15.5	-4.40	DMN
		Clus 4	R. Frontal_Sup_Medial	53	4.5, 53.5, 13.5	-3.83	DMN
		Clus 5	L. Frontal_Sup_Medial	23	-7.5, 57.5, 19.5	-3.75	DMN
		Clus 6	R. Angular	126	42.5, -50.5, 23.5	-5.13	DMN
		Clus 7	R. Frontal_Sup_Medial	48	4.5, 65.5, 23.5	-4.49	DMN
		Clus 8	R. Precuneus	68	14.5, -48.5, 23.5	-4.15	DMN
		Clus 9	L. Cuneus	62	-15.5, -56.5, 27.5	-4.53	DMN
		Clus 0	R. Precuneus	213	6.5, -62.5, 39.5	-4.40	DMN
**R. dACC**	SN	Clus 1	L. Lingual	20	-15.5, -94.5, -18.5	-3.97	VN
		Clus 2	L. Hippocampus	74	-27.5, -28.5, -4.5	-5.03	
		Clus 3	R. Precuneus	23	8.5, -60.5, 33.5	-3.49	DMN
**L. dACC**	SN	Clus 1	R. Temporal_Mid	31	56.5, 9.5, -26.5	-4.15	DMN
		Clus 2	R. Hippocampus	32	34.5, -8.5, -20.5	-4.23	
		Clus 3	L. Frontal_Inf_Orb	25	-47.5, 29.5, -12.5	-4.10	DMN
		Clus 4	L. Hippocampus	22	-29.5, -28.5, -4.5	-3.97	
		Clus 5	L. Frontal_Sup_Medial	62	-1.5, 63.5, 15.5	-4.38	DMN
		Clus 6	R. Angular	20	48.5, -74.5, 37.5	-4.17	DMN
**L. vlPFC**	SN	Clus 1	L. Frontal_Inf_Oper	48	-43.5, 3.5, 25.5	3.82	DAN
**R. vlPFC**	SN	Clus 1	L. Temporal_Inf	28	-53.5, -26.5, -26.5	-3.97	SCN
		Clus 2	R. Frontal_Mid	22	38.5, 3.5, 61.5	3.87	CEN

For seeds in the DMN, BD patients showed extensive decreased FCs within the DMN compared to HCs. Decreased FC was observed in BD patients between the bilateral vmPFC and extensive regions within the DMN. In addition, these regions included the bilateral ACC, right angular gyrus (AG), right middle part of the SFG and right precuneus. The left vmPFC showed extra decreased FCs with the left middle part of the SFG and left cuneus, whereas the right vmPFC showed decreased FC with the left precuneus in BD patients. Moreover, decreased FCs within the DMN were also observed between the right PCC seed and left ACC, as well as between the left PCC seed and right ACC/right superior temporal gyrus. BD patients also exhibited decreased FC between the left PCC and the triangular part of the IFG in the SN (Table 2).

For seeds in the SN, most of the altered FCs involved the DMN. Specifically, decreased FCs were observed in BD patients between the right dACC and right precuneus, as well as between the left dACC and right AG/anterior parts of the DMN (i.e., the left medial part of the SFG, left orbital part of the IFG and right middle temporal gyrus). Moreover, BD patients demonstrated increased FC between the right vlPFC and right MFG in the CEN (Table 2).

### Network-wise connections

From the perspective of network-wise connections, four pairs of FCs fell into CEN-SN connections, thus indicating increased FCs between CEN and SN. BD patients exhibited decreased connections between the DMN and SN (six FCs), between the DMN and CEN (two FCs) and within the DMN (18 FCs) (Fig. 1B).

### Relationship between functional connectivity and neuropsychological assessments

**Fig. 2:**
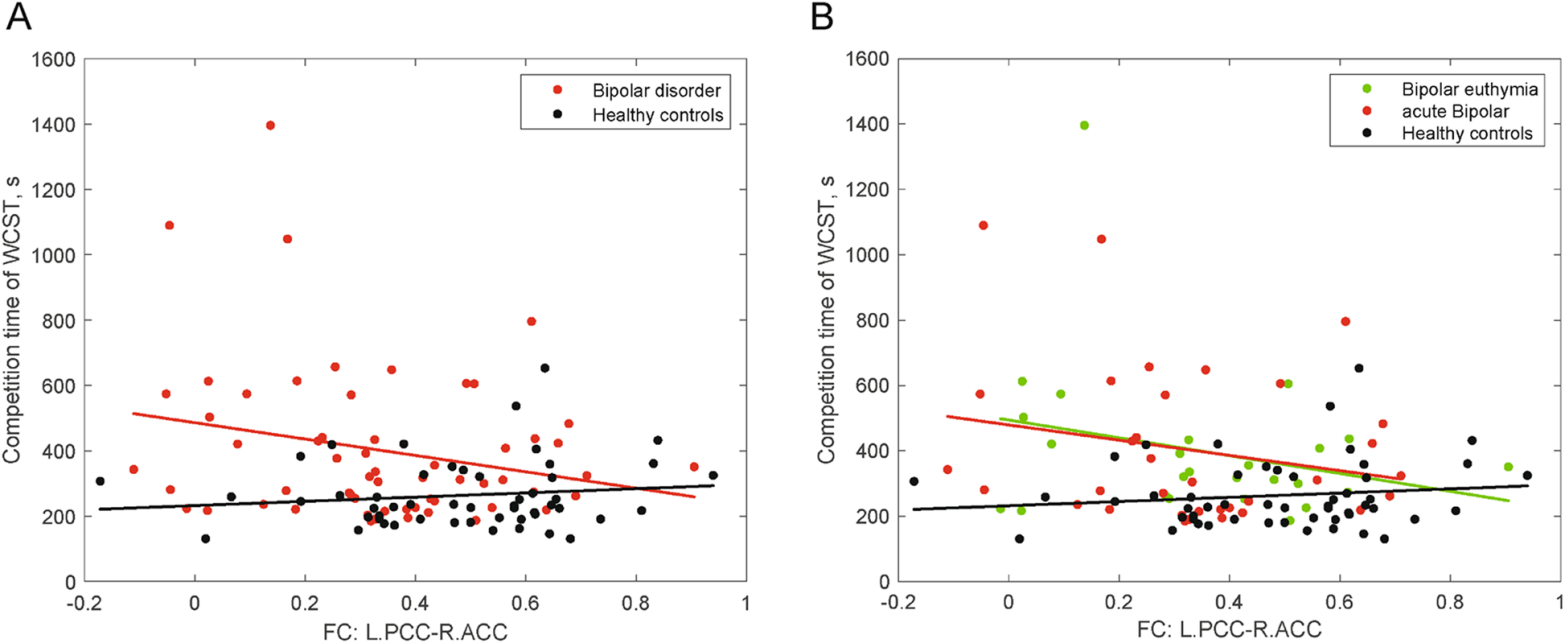
Different associations between functional connectivity and diagnostic groups for cognitive performance

A significant diagnostic-group-by-left PCC-right ACC FC interaction was observed for WCST completion time (overall model fit: *p* < 0.001). The significant diagnostic-group-by-FC interaction (*t* = 2.265, *p* = 0.026) was due to a negative relationship between WCST completion time and left PCC-right ACC FC in BD patients (standardized *β* = -0.356, *p* = 0.01) but a nonsignificant relationship in HCs (standardized *β* = 0.089, *p* = 0.563) after controlling for age, sex, years of education and head motion (Fig. 2A).

### Sensitivity analysis

Twenty-nine of the thirty significant FCs in comparisons between BD patients and HCs showed group differences in comparisons between euthymic patients with BD and HCs (Table 3).

**Table 3:** Functional connectivity differences related to the three core neurocognitive networks between euthymic patients with BD and HCs

Seed	Target network	Cluster	Clusters which showed significant FC with seed	*t* value	*p* value	Network
**L. IPL**	CEN	Clus 2	L. Frontal_Sup_Orb	-3.06*	0.003	DMN
		Clus 3	R. Insula	4.07*	1.20 × 10^–4^	SN
		Clus 4	L. Rolandic_Oper	3.91*	2.12 × 10^–4^	SN
		Clus 7	L. Frontal_Mid	-2.76*	0.007	DMN
		Clus 8	R. Paracentralobule	4.37*	4.19 × 10^–5^	SN
**R. vmPFC**	DMN	Clus 2	R. Cingulum_Ant	-4.07*	1.22 × 10^–4^	DMN
		Clus 3	L. Cingulum_Ant	-3.81*	2.93 × 10^–4^	DMN
		Clus 4	R. Angular	-2.99*	0.004	DMN
		Clus 5	R. Frontal_Sup_Medial	-2.90*	0.005	DMN
		Clus 6	L. Precuneus	-3.52*	7.68 × 10^–4^	DMN
		Clus 7	R. Precuneus	-2.98*	0.004	DMN
**R. PCC**	DMN	Clus 4	L. Cingulum_Ant	-3.33*	0.001	DMN
**L. PCC**	DMN	Clus 1	R. Frontal_Inf_Tri	-2.95*	0.004	SN
		Clus 2	R. Cingulum_Ant	-2.83*	0.006	DMN
		Clus 3	R. Temporal_Sup	-2.41*	0.019	DMN
**L. vmPFC**	DMN	Clus 2	R. Cingulum_Ant	-3.94*	1.90 × 10^–4^	DMN
		Clus 3	L. Cingulum_Ant	-3.75*	3.57 × 10^–4^	DMN
		Clus 4	R. Frontal_Sup_Medial	-2.67*	0.009	DMN
		Clus 5	L. Frontal_Sup_Medial	-1.17	0.244	DMN
		Clus 6	R. Angular	-3.23*	0.002	DMN
		Clus 7	R. Frontal_Sup_Medial	-2.69*	0.009	DMN
		Clus 8	R. Precuneus	-3.07*	0.003	DMN
		Clus 9	L. Cuneus	-3.49*	8.26 × 10^–4^	DMN
		Clus 0	R. Precuneus	-2.99*	0.004	DMN
**R. dACC**	SN	Clus 3	R. Precuneus	-2.47*	0.016	DMN
**L. dACC**	SN	Clus 1	R. Temporal_Mid	-2.77*	0.007	DMN
		Clus 3	L. Frontal_Inf_Orb	-2.20*	0.031	DMN
		Clus 5	L. Frontal_Sup_Medial	-2.77*	0.007	DMN
		Clus 6	R. Angular	-2.26*	0.027	DMN
**R. vlPFC**	SN	Clus 2	R. Frontal_Mid	2.79*	0.007	CEN

*significant after FDR correction

No significant relationship was found between WCST completion time and left PCC-right ACC FC (Fig. 2B) in euthymic patients with BD (standardized *β* = -0.462, *p* = 0.058) and in acute patients with BD (standardized *β* = -0.313, *p* = 0.099). After including current mood state as one of predictors, a negative relationship was found between WCST completion time and left PCC-right ACC FC in BD patients (standardized *β* = -0.355, *p* = 0.012). However, the summary analysis of variance (ANOVA) with an *F* test for the model was not significant (overall model fit: *p* < 0.104).

After including a binary variable indicating medication status as one of predictors, a negative relationship between WCST completion time and left PCC-right ACC FC was also found in BD patients (standardized *β* = -0.361, *p* = 0.008; overall model fit: *p* < 0.026).

## Discussion

Our primary finding was that BD patients showed extensive decreased functional connectivity within the DMN at both the regional and network levels and less extensive abnormal connections in the DMN-SN, DMN-CEN and SN-CEN. Specifically, we observed decreased functional connectivity between the DMN and SN and between the DMN and CEN but increased functional connectivity between the SN and CEN. Consistent with our hypothesis, a different relationship between PCC-ACC FC and executive function performance was observed in BD patients and HCs. Furthermore, compared with HCs, BD patients showed decreased functional connectivity between the PCC and ACC. Reduced PCC-ACC FC was solely associated with worse cognitive performances in BD patients (but not in HCs).

Cognitive deficits in BD have received much interest for years. Substantial evidence suggests that larger deficits in executive function and sustained attention are present in euthymic patients with BD [32, 33]. Interestingly, symptom fluctuations do not seem to explain such cognitive deficits [34]. Such cognitive deficits are persistent and stable over time, especially impaired sustained attention [35–37]. Consistent with this extant evidence, our results showed that BD patients exhibited more pronounced dysfunction in attention domain, in terms of the number and effect size of neuropsychological measures. Our results also revealed reduced PCC-ACC FC was associated with longer WCST completion time, where the WCST is used to assess cognitive flexibility, one of the three core functions of executive function [38]. Supportive evidence from another study employing dynamic functional connectivity reported that a decreased variability in the dynamic FC between medial prefrontal cortex and PCC was associated with reduced cognitive set-shifting [39]. Thus, Massalha et al. [20] proposed that the two hubs of the DMN, i.e., the vmPFC/ACC and PCC, may underlie executive function deficits in BD.

Our findings regarding the resting-state FC of the three core neurocognitive networks at the region level emphasize the eminent role of anterior cortical midline structures in BD. Specifically, our analysis at the regional level demonstrated substantially decreased vmPFC-based and dACC-based connectivity, mostly with regions in the DMN. In parallel with our findings, several studies have reported reduced medial prefrontal cortex-based FC [8, 40], particularly with the rest of the frontal cortex [41] and other regions of the DMN, such as the PCC [42], in BD. Reduced functional connectivity between the ACC and IFG was previously reported in BD patients during the Stroop Colour Word Task, thus indicating a poorer ability to minimize resources devoted to monitoring internal states in the service of task demands [43]. Another study found that FC between supragenual ACC and perigenual ACC was associated with processing speed in CPT [18]. Wang et al. reported altered amplitude of low frequency fluctuation in precuneus and ACC in BD patients, which was positively associated with processing speed in the Symbol Coding test [44]. Notably, recent evidence has suggested that higher serum inflammatory marker levels are associated with decreased FC among anterior cortical midline structures [45], indicating that elevated inflammatory responses may contribute to the pathophysiology of cognitive deficits in BD [9]. Taken together, these findings lend support to the role of resting-state functional connectivity of the anterior cortical midline structures in broad cognitive deficits in BD patients, and the immune response may be the underlying mechanisms.

In terms of resting-state FC of the three core neurocognitive networks at the network level, extant findings are conflicting, particularly in terms of internetwork SN-CEN and SN-DMN FC. Consistent with our findings, one ICA-based study observed hyperconnectivity in SN-CEN in euthymic BD [46]. The opposite finding was observed in another study that was conducted in depressive subjects, in which bipolar depression was associated with decreased insular-IPL FC [30]. Despite the heterogeneity of the included states of the illness, different approaches, such as dynamic FC [47] and task-related FC [48], may also contribute to the conflicting findings. For SN-DMN connectivity probing, decreased FC was observed between the dACC and anterior/posterior DMN in our study and between the sgACC and PCC in another study by Rey et al. [49]. However, increased FC was observed between the insular and anterior/posterior DMN [40, 50] and hippocampus [51]. These findings suggest that there are deviations in coordinated connectivity within the SN, particularly between the sgACC/dACC and insula. Consistently, hyperconnectivity between the insula and IPL was observed in our study. In terms of CEN-DMN connectivity probing, although only two altered connectivities were observed between the CEN and DMN in our study, the alteration direction was quite consistent with that of previous studies [8, 40, 50, 52].

The findings of decreased FC within the DMN and in DMN-SN and DMN-CEN (but increased FC in SN-CEN) suggest that there is less coordinated connectivity within the DMN and between the DMN and other brain networks. Consistently, many previous studies have demonstrated decreased connectivity between the anterior and posterior DMN in BD [7, 18, 53]. Another study also reported an elevated FC in SN-CEN in BD patients and the authors speculated that the alterations may reflect a greater assignment of saliency to external stimuli, contributing to sustained attention deficits in BD [40]. Interestingly, this hypoconnectivity has also been observed in ADHD, which is characterized by distractibility and hyperactivity [54]. In particular, longitudinal studies suggest that a persistent attention deficit in BD, irrespective of manic and depressive symptoms fluctuating, may be a state-modulated but trait marker for BD [34, 36, 55]. The decreased connectivity between the DMN and SN, as well as the increased connectivity between the CEN and SN, may reflect a reduced salience attribution to internal stimuli and a greater assignment of saliency to external stimuli, respectively [3, 4]. This scenario may hypothetically result in stable attention deficit in BD, which simultaneously serve as a cognitive endophenotype and a vulnerability marker [56].

The observed group differences in the relationship between the anterior to posterior DMN FC and WCST completion time may present maladaptive changes in BD patients. Although no significant relationships were observed between PCC-ACC FC and WCST completion time in HCs, it is notable that this connectivity is disrupted with age, thus accounting for the vulnerability to cognitive decline [57]. In our study, a similar positive association between connectivity strength and executive function suggests the possibility of an underlying accelerated ageing process in BD [39]. The hypoconnectivity between the PCC and ACC in BD may suggest a worse function of abstraction and mental flexibility [58] similar to ageing adults. Moreover, it is worth noting that anterior to posterior DMN FC variability has also been found to be associated with processing speed and cognitive set-shifting function [39].

Several limitations of this study should be acknowledged. First, several studies have examined the contribution of mood episodes to brain function [59–62], but the interaction effect of subgroup and brain function on cognitive performance has not yet been investigated. Despite the effort to examine the impact of current mood state on relationship between FC and neuropsychological assessments in BD patients, the relationship between WCST completion time and left PCC-right ACC FC was not observed in acute or euthymic patients with BD and the model with current mood state as covariate was not significant, which makes it difficult to decipher the effects of current mood episode on functional connectivity and cognitive performance. The modest sample size and unbalanced sample size of the subgroups in our study may be attributed to the nonsignificant finding. Specific study designs, especially longitudinal designs, are needed in future studies [63]. Second, psychotropic medications could be a possible confounding factor. Theoretically, psychotropic treatment is associated with brain function alterations [64], but this study design fails to identify the medication effects. Third, although resting-state functional connectivity provides a useful tool for examining brain activity underlying cognitive performance, task-related activation during cognitive tasks can complement our understanding of neural correlates that underly cognitive deficits in BD.

## Conclusions

In summary, our study emphasizes the dominant role of the DMN in the triple brain networks that are implicated in the psychopathology of BD. Specifically, the findings of decreased DMN-CEN and DMN-SN and within DMN FC (but increased SN-CEN) may suggest a potential adaptive change in communications of the DMN with other networks promoted by the incongruous intrinsic activity of the DMN. Moreover, connectivity patterns between the anterior and posterior DMN are differentially associated with executive function performance between individuals with and without BD. This different relationship between intrinsic DMN connectivity and cognitive performance between diagnostic groups provides evidence that future neuromodulation therapy for cognitive deficits may benefit from the consideration of the DMN as a potential target.

## Data Availability

The dataset is not publicly available due to privacy and ethical restrictions but is available from the corresponding author on reasonable request.

## Abbreviations

BD: Bipolar disorder
HC: Healthy control
FC: Functional connectivity
DMN: Default mode network
CEN: Central executive network
SN: Salience network
HRSD: Hamilton Rating Scale for Depression
YMRS: Young Mania Rating Scale
PANAS: Positive And Negative Affect Schedule
WCST: Wisconsin Card Sorting Test
CPT: Continuous Performance Test
PCC: Posterior cingulate cortex
vmPFC: Ventromedial prefrontal cortex
ACC: Anterior cingulate cortex
dACC: Dorsal anterior cingulate cortex
sgACC: Subgenual anterior cingulate cortex
IPL: Inferior parietal lobule
vlPFC: Ventrolateral prefrontal cortex
dlPFC: Dorsolateral prefrontal cortex
IFG: Inferior frontal gyrus
SFG: Superior frontal gyrus
MFG: Left middle frontal gyrus
AG: Angular gyrus
M.I.N.I.: Mini-International Neuropsychiatric Interview
MRI: Magnetic resonance imaging
PA: Positive affect
NA: Negative affect
MNI: Montreal Neurological Institute
FD: Framewise displacement

## References

[1] Bourne C, Aydemir O, Balanza-Martinez V, Bora E, Brissos S, Cavanagh JT (2013). Neuropsychological testing of cognitive impairment in euthymic bipolar disorder: an individual patient data meta-analysis. Acta Psychiatr Scand.

[2] Miskowiak KW, Carvalho AF, Vieta E, Kessing LV (2016). Cognitive enhancement treatments for bipolar disorder: A systematic review and methodological recommendations. Eur Neuropsychopharmacol.

[3] Menon V (2011). Large-scale brain networks and psychopathology: a unifying triple network model. Trends Cogn Sci.

[4] Menon V, Uddin LQ (2010). Saliency, switching, attention and control: a network model of insula function. Brain Struct Funct.

[5] Townsend J, Altshuler LL (2012). Emotion processing and regulation in bipolar disorder: a review. Bipolar Disord.

[6] Mason L, Eldar E, Rutledge RB (2017). Mood Instability and Reward Dysregulation-A Neurocomputational Model of Bipolar Disorder. JAMA Psychiat.

[7] Zovetti N, Rossetti MG, Perlini C, Maggioni E, Bontempi P, Bellani M (2020). Default mode network activity in bipolar disorder. Epidemiol Psychiatr Sci.

[8] Yoon S, Kim TD, Kim J, Lyoo IK (2021). Altered functional activity in bipolar disorder: A comprehensive review from a large-scale network perspective. Brain Behav.

[9] Tseng HH, Chang HH, Wei SY, Lu TH, Hsieh YT, Yang YK (2021). Peripheral inflammation is associated with dysfunctional corticostriatal circuitry and executive dysfunction in bipolar disorder patients. Brain Behav Immun.

[10] Zarp Petersen J, Varo C, Skovsen CF, Ott CV, Kjaerstad HL, Vieta E (2022). Neuronal underpinnings of cognitive impairment in bipolar disorder: A large data-driven functional magnetic resonance imaging study. Bipolar Disord.

[11] Sepede G, De Berardis D, Campanella D, Perrucci MG, Ferretti A, Serroni N (2012). Impaired sustained attention in euthymic bipolar disorder patients and non-affected relatives: an fMRI study. Bipolar Disord.

[12] Pomarol-Clotet E, Alonso-Lana S, Moro N, Sarro S, Bonnin MC, Goikolea JM (2015). Brain functional changes across the different phases of bipolar disorder. Br J Psychiatry.

[13] Alonso-Lana S, Moro N, McKenna PJ, Sarro S, Romaguera A, Monte GC (2019). Longitudinal brain functional changes between mania and euthymia in bipolar disorder. Bipolar Disord.

[14] Dell'Osso B, Cinnante C, Di Giorgio A, Cremaschi L, Palazzo MC, Cristoffanini M (2015). Altered prefrontal cortex activity during working memory task in Bipolar Disorder: A functional Magnetic Resonance Imaging study in euthymic bipolar I and II patients. J Affect Disord.

[15] Ajilore O, Vizueta N, Walshaw P, Zhan L, Leow A, Altshuler LL (2015). Connectome signatures of neurocognitive abnormalities in euthymic bipolar I disorder. J Psychiatr Res.

[16] Townsend JD, Bookheimer SY, Foland-Ross LC, Moody TD, Eisenberger NI, Fischer JS (2012). Deficits in inferior frontal cortex activation in euthymic bipolar disorder patients during a response inhibition task. Bipolar Disord.

[17] Strakowski SM, Adler CM, Holland SK, Mills N, DelBello MP (2004). A preliminary FMRI study of sustained attention in euthymic, unmedicated bipolar disorder. Neuropsychopharmacology.

[18] Magioncalda P, Martino M, Conio B, Escelsior A, Piaggio N, Presta A (2015). Functional connectivity and neuronal variability of resting state activity in bipolar disorder–reduction and decoupling in anterior cortical midline structures. Hum Brain Mapp.

[19] Yu H, Li ML, Meng Y, Li XJ, Wei W, Li YF (2021). Inferior frontal gyrus seed-based resting-state functional connectivity and sustained attention across manic/hypomanic, euthymic and depressive phases of bipolar disorder. J Affect Disord.

[20] Massalha Y, Maggioni E, Callari A, Brambilla P, Delvecchio G (2023). A review of resting-state fMRI correlations with executive functions and social cognition in bipolar disorder. J Affect Disord.

[21] Albert KM, Potter GG, Boyd BD, Kang H, Taylor WD (2019). Brain network functional connectivity and cognitive performance in major depressive disorder. J Psychiatr Res.

[22] Sheffield JM, Rogers BP, Blackford JU, Heckers S, Woodward ND (2019). Accelerated Aging of Functional Brain Networks Supporting Cognitive Function in Psychotic Disorders. Biol Psychiat.

[23] Sheehan DV, Lecrubier Y, Sheehan KH, Amorim P, Janavs J, Weiller E (1998). The Mini-International Neuropsychiatric Interview (M.I.N.I.): the development and validation of a structured diagnostic psychiatric interview for DSM-IV and ICD-10. J Clin Psychiatry.

[24] Hamilton M (1960). A rating scale for depression. J Neurol Neurosurg Psychiatry.

[25] Young RC, Biggs JT, Ziegler VE, Meyer DA (1978). A rating scale for mania: reliability, validity and sensitivity. Br J Psychiatry.

[26] Watson D, Clark LA, Tellegen A (1988). Development and validation of brief measures of positive and negative affect: the PANAS scales. J Pers Soc Psychol.

[27] Conners CK, Epstein JN, Angold A, Klaric J (2003). Continuous performance test performance in a normative epidemiological sample. J Abnorm Child Psychol.

[28] Rk H (1981). The Wisconsin Card Sorting Test Manual.

[29] Zhang YY, Brady M, Smith S (2001). Segmentation of brain MR images through a hidden Markov random field model and the expectation-maximization algorithm. Ieee T Med Imaging.

[30] Ellard KK, Zimmerman JP, Kaur N, Van Dijk KRA, Roffman JL, Nierenberg AA (2018). Functional Connectivity Between Anterior Insula and Key Nodes of Frontoparietal Executive Control and Salience Networks Distinguish Bipolar Depression From Unipolar Depression and Healthy Control Subjects. Biol Psychiatry Cogn Neurosci Neuroimaging.

[31] Eklund A, Nichols TE, Knutsson H (2016). Cluster failure: Why fMRI inferences for spatial extent have inflated false-positive rates. Proc Natl Acad Sci USA.

[32] Kurtz MM, Gerraty RT (2009). A meta-analytic investigation of neurocognitive deficits in bipolar illness: profile and effects of clinical state. Neuropsychology.

[33] Arts B, Jabben N, Krabbendam L, van Os J (2008). Meta-analyses of cognitive functioning in euthymic bipolar patients and their first-degree relatives. Psychol Med.

[34] Lima IMM, Peckham AD, Johnson SL (2018). Cognitive deficits in bipolar disorders: Implications for emotion. Clin Psychol Rev.

[35] Chaves OC, Lombardo LE, Bearden CE, Woolsey MD, Martinez DM, Barrett JA (2011). Association of clinical symptoms and neurocognitive performance in bipolar disorder: a longitudinal study. Bipolar Disord.

[36] Santos JL, Aparicio A, Bagney A, Sanchez-Morla EM, Rodriguez-Jimenez R, Mateo J (2014). A five-year follow-up study of neurocognitive functioning in bipolar disorder. Bipolar Disord.

[37] Maalouf FT, Klein C, Clark L, Sahakian BJ, Labarbara EJ, Versace A (2010). Impaired sustained attention and executive dysfunction: bipolar disorder versus depression-specific markers of affective disorders. Neuropsychologia.

[38] Miles S, Howlett CA, Berryman C, Nedeljkovic M, Moseley GL, Phillipou A (2021). Considerations for using the Wisconsin Card Sorting Test to assess cognitive flexibility. Behav Res Methods.

[39] Nguyen TT, Kovacevic S, Dev SI, Lu K, Liu TT, Eyler LT (2017). Dynamic functional connectivity in bipolar disorder is associated with executive function and processing speed: A preliminary study. Neuropsychology.

[40] Wang J, Wang Y, Wu X, Huang H, Jia Y, Zhong S (2020). Shared and specific functional connectivity alterations in unmedicated bipolar and major depressive disorders based on the triple-network model. Brain Imaging Behav.

[41] Anticevic A, Brumbaugh MS, Winkler AM, Lombardo LE, Barrett J, Corlett PR (2013). Global prefrontal and fronto-amygdala dysconnectivity in bipolar I disorder with psychosis history. Biol Psychiat.

[42] Gong J, Chen G, Jia Y, Zhong S, Zhao L, Luo X (2019). Disrupted functional connectivity within the default mode network and salience network in unmedicated bipolar II disorder. Prog Neuropsychopharmacol Biol Psychiatry.

[43] Pompei F, Dima D, Rubia K, Kumari V, Frangou S (2011). Dissociable functional connectivity changes during the Stroop task relating to risk, resilience and disease expression in bipolar disorder. Neuroimage.

[44] Wang Z, Meda SA, Keshavan MS, Tamminga CA, Sweeney JA, Clementz BA (2015). Large-Scale Fusion of Gray Matter and Resting-State Functional MRI Reveals Common and Distinct Biological Markers across the Psychosis Spectrum in the B-SNIP Cohort. Front Psychiatry.

[45] Tu PC, Li CT, Lin WC, Chen MH, Su TP, Bai YM (2017). Structural and functional correlates of serum soluble IL-6 receptor level in patients with bipolar disorder. J Affect Disord.

[46] Das P, Calhoun V, Malhi GS (2014). Bipolar and borderline patients display differential patterns of functional connectivity among resting state networks. Neuroimage.

[47] Pang Y, Chen H, Wang Y, Long Z, He Z, Zhang H (2018). Transdiagnostic and diagnosis-specific dynamic functional connectivity anchored in the right anterior insula in major depressive disorder and bipolar depression. Prog Neuropsychopharmacol Biol Psychiatry.

[48] Ellard KK, Gosai AK, Felicione JM, Peters AT, Shea CV, Sylvia LG (2019). Deficits in frontoparietal activation and anterior insula functional connectivity during regulation of cognitive-affective interference in bipolar disorder. Bipolar Disord.

[49] Rey G, Piguet C, Benders A, Favre S, Eickhoff SB, Aubry JM (2016). Resting-state functional connectivity of emotion regulation networks in euthymic and non-euthymic bipolar disorder patients. Eur Psychiatry.

[50] Chai XJ, Whitfield-Gabrieli S, Shinn AK, Gabrieli JD, Nieto Castanon A, McCarthy JM (2011). Abnormal medial prefrontal cortex resting-state connectivity in bipolar disorder and schizophrenia. Neuropsychopharmacology.

[51] Li J, Tang Y, Womer F, Fan G, Zhou Q, Sun W (2018). Two patterns of anterior insular cortex functional connectivity in bipolar disorder and schizophrenia. World J Biol Psychiatry.

[52] Favre P, Baciu M, Pichat C, Bougerol T, Polosan M (2014). fMRI evidence for abnormal resting-state functional connectivity in euthymic bipolar patients. J Affect Disord.

[53] Wang J, Wang Y, Wu X, Huang H, Jia Y, Zhong S, et al. Shared and specific functional connectivity alterations in unmedicated bipolar and major depressive disorders based on the triple-network model. Brain imaging and behavior. 2018:10.1007/s11682-018-9978-x. 10.1007/s11682-018-9978-x 30382529

[54] Uddin LQ, Kelly AM, Biswal BB, Castellanos FX, Milham MP (2009). Functional connectivity of default mode network components: correlation, anticorrelation, and causality. Hum Brain Mapp.

[55] Clark L, Goodwin GM (2004). State- and trait-related deficits in sustained attention in bipolar disorder. Eur Arch Psychiatry Clin Neurosci.

[56] Bora E, Yucel M, Pantelis C (2009). Cognitive endophenotypes of bipolar disorder: a meta-analysis of neuropsychological deficits in euthymic patients and their first-degree relatives. J Affect Disord.

[57] Andrews-Hanna JR, Snyder AZ, Vincent JL, Lustig C, Head D, Raichle ME (2007). Disruption of large-scale brain systems in advanced aging. Neuron.

[58] Volkow ND, Gur RC, Wang GJ, Fowler JS, Moberg PJ, Ding YS (1998). Association between decline in brain dopamine activity with age and cognitive and motor impairment in healthy individuals. Am J Psychiatry.

[59] Li M, Huang C, Deng W, Ma X, Han Y, Wang Q (2015). Contrasting and convergent patterns of amygdala connectivity in mania and depression: a resting-state study. J Affect Disord.

[60] Russo D, Martino M, Magioncalda P, Inglese M, Amore M, Northoff G. Opposing changes in the functional architecture of large-scale networks in bipolar mania and depression. Schizophr Bull. 2020;46(4):971–80. 10.1093/schbul/sbaa004 PMC7342167 32047938

[61] Zhang L, Li W, Wang L, Bai T, Ji GJ, Wang K (2020). Altered functional connectivity of right inferior frontal gyrus subregions in bipolar disorder: a resting state fMRI study. J Affect Disord.

[62] Martino M, Magioncalda P, Huang Z, Conio B, Piaggio N, Duncan NW (2016). Contrasting variability patterns in the default mode and sensorimotor networks balance in bipolar depression and mania. Proc Natl Acad Sci USA.

[63] Phillips ML, Swartz HA (2014). A critical appraisal of neuroimaging studies of bipolar disorder: toward a new conceptualization of underlying neural circuitry and a road map for future research. Am J Psychiatry.

[64] Phillips ML, Travis MJ, Fagiolini A, Kupfer DJ (2008). Medication effects in neuroimaging studies of bipolar disorder. Am J Psychiatry.

